# Small molecule receptor tyrosine kinase inhibitor of platelet-derived growth factor signaling (SU9518) modifies radiation response in fibroblasts and endothelial cells

**DOI:** 10.1186/1471-2407-6-79

**Published:** 2006-03-24

**Authors:** Minglun Li, Gong Ping, Christian Plathow, Thuy Trinh, Kenneth E Lipson, Kai Hauser, Robert Krempien, Juergen Debus, Amir Abdollahi, Peter E Huber

**Affiliations:** 1 Department of Radiation Oncology, German Cancer Research Centre (DKFZ), Heidelberg, Germany; 2 University of Heidelberg Medical School, Heidelberg, Germany; 3 Department of Clinical Radiology, University Hospital Heidelberg, Germany; 4 SUGEN, Inc., South San Francisco, California 94080-4811, USA; 5 Department of Radiation Oncology, University Hospital Tuebingen, Germany; 6 Department of Diagnostic Radiology, University Hospital Tuebingen, Germany; 7 Institucio Catalana de Recerca i Estudis Avancats (ICREA), Barcelona, Spain; 8 Department of Mathematics, University of California, Berkeley, CA, USA

## Abstract

**Background:**

Several small receptor tyrosine kinase inhibitors (RTKI) have entered clinical cancer trials alone and in combination with radiotherapy or chemotherapy. The inhibitory spectrum of these compounds is often not restricted to a single target. For example Imatinib/Gleevec (primarily a bcr/abl kinase inhibitor) or SU11248 (mainly a VEGFR inhibitor) are also potent inhibitors of PDGFR and other kinases. We showed previously that PDGF signaling inhibition attenuates radiation-induced lung fibrosis in a mouse model. Here we investigate effects of SU9518, a PDGFR inhibitor combined with ionizing radiation in human primary fibroblasts and endothelial cells *in vitro*, with a view on utilizing RTKI for antifibrotic therapy.

**Methods:**

Protein levels of PDGFR-α/-β and phosphorylated PDGFR in fibroblasts were analyzed using western and immunocytochemistry assays. Functional proliferation and clonogenic assays were performed (i) to assess PDGFR-mediated survival and proliferation in fibroblasts and endothelial cells after SU9518 (small molecule inhibitor of PDGF receptor tyrosine kinase); (ii) to test the potency und selectivity of the PDGF RTK inhibitor after stimulation with PDGF isoforms (-AB, -AA, -BB) and VEGF+bFGF. In order to simulate *in vivo *conditions and to understand the role of radiation-induced paracrine PDGF secretion, co-culture models consisting of fibroblasts and endothelial cells were employed.

**Results:**

In fibroblasts, radiation markedly activated PDGF signaling as detected by enhanced PDGFR phosphorylation which was potently inhibited by SU9518. In fibroblast clonogenic assay, SU9518 reduced PDGF stimulated fibroblast survival by 57%. Likewise, SU9518 potently inhibited fibroblast and endothelial cell proliferation. In the co-culture model, radiation of endothelial cells and fibroblast cells substantially stimulated proliferation of non irradiated fibroblasts and vice versa. Importantly, the RTK inhibitor significantly inhibited this paracrine radiation-induced fibroblast and endothelial cell activation.

**Conclusion:**

Radiation-induced autocrine and paracrine PDGF signaling plays an important role in fibroblast and endothelial cell proliferation. SU9518, a PDGFR tyrosine kinase inhibitor, reduces radiation-induced fibroblast and endothelial cell activation. This may explain therapeutic anticancer effects of Imatinib/Gleevec, and at the same time it could open a way of attenuating radiation-induced fibrosis.

## Background

The development of fibrosis is a frequent side effect that may be caused by a variety of inductors. Fibrosis especially hampers cancer treatment such as radiotherapy and chemotherapy, thus severely limiting cancer therapy success. Furthermore, the development of fibrosis may reflect the integrative and interdependent roles of various cell compounds in tumor biology calling into question reductionist approaches focus on individual tumor cell compartment. Fibrosis occurs in many organs. Lung fibrosis for example may be caused by radiotherapy, chemotherapeutic drugs (such as bleomycin), amiodaron or, chemical substances (like asbestos fibers and silica particles, etc [1]). With the progression of the disease patients develop severe clinical symptoms limiting oxygenation of the blood and patients' survival. Treatment of fibrosis remains elusive given that the exact mediators and mechanisms involved in fibrogenesis are unknown [2,3].

Fibrosis is characterized by the excessive proliferation of mesenchymal cells (fibroblasts, myofibroblasts, and smooth muscle cells) and the subsequent deposition of extracellular matrix proteins [2]. Fibroblasts exhibit increased chemotaxis, proliferation, and extracellular matrix production in fibrotic lung and skin [2,4]. Cytokines such as platelet-derived growth factor (PDGF), transforming growth factor (TGF)-α and -β, interleukin (IL)-1α and -β, and basic fibroblast growth factor (bFGF) have emerged as major stimulators of the fibroproliferative process induced by diverse stimuli such as ionizing radiation [5-7].

Here we focus on PDGF which has been implicated in a wide variety of pathological processes, including pulmonary fibrosis and skin fibrosis [8-10]. PDGF is a disulfide-linked dimer of two related polypeptide chains, designated A, B, C and D, which are assembled as heterodimers (PDGF-AB) or homodimers (PDGF-AA, PDGF-BB, PDGF-CC and PDGF-DD) [11]. PDGF exerts its biological activity by binding to structurally similar PDGF receptors (PDGFR-α and -β). It induces receptor dimerization and autophosphorylation of the receptor tyrosine kinase (RTK). Activated RTK phosphorylates numerous signaling molecules that initiate the intracellular signaling cascades leading to cell proliferation and survival [12]. These phosphorylation-dependent interactions are essential for the activation of intracellular signaling pathways that can lead to tissue fibrosis. It seems reasonable to assume that blocking phosphorylation of PDGFR could be a way of preventing biological effects of this cytokine and thus fibrogenesis.

Recently, we have demonstrated this concept *in vivo *in a radiation-induced lung fibrosis model in mice by showing that inhibition of PDGF signaling functionally attenuates pulmonary fibrosis [13]. Here we analyze the underlying mechanistic cell effects *in vitro *with respect to the cellular pathway of RTK inhibition and to radiation for the PDGF/PDGFR system in human primary fibroblasts and endothelial cells.

## Materials and methods

### Endothelial and fibroblast cell cultures and reagents

Primary isolated human dermal microvascular vein endothelial cells (HDMVEC, Promocell, Heidelberg, Germany) and primary isolated human fibroblasts (Promocell, Heidelberg, Germany) were cultured up to passage 6. Cells were maintained in culture at 37°C with 5% CO_2 _and 95% humidity in serum reduced (5% fetus calf serum) modified promocell media (MPM) supplemented with 2 ng/ml vascular endothelial growth factor (VEGF) and 4 ng/ml basic fibroblast growth factor (bFGF). This combination of VEGF and bFGF optimized growth kinetics [14]. Human recombinant VEGF and bFGF proteins were purchased from Promocell.

### Western blotting

Primary human lung fibroblasts were grown in modified media supplemented with 5% fetus calf serum containing 2 ng/ml VEGF and 4 ng/ml bFGF in 125 cm^2 ^flasks. Cells were then treated either with 10 Gy or 5 μM SU9518 exclusively or in combination with both (1 hour SU9518 incubation prior to radiation). 1/2 hour after radiation fibroblasts were washed twice with ice-cold PBS, lysed with 250 μl ice-cold cell lysis buffer (1 M Tris, 5 M NaCl, 1 mM EDTA, 1 mM EGTA, pH 8.01). Lysates were kept on ice, and centrifuged at 4°C for 10 minutes at 13000 rpm. Supernatant was collected for western blotting assay as described [15]. Proteins were transferred from a polyacrylamide gel to a nitrocellulose membrane with a Mini-Trans-Blotter (100 V for 90 minutes) in transfer buffer. After transfer, non-specific binding sites were blocked for 1 hour with 5% non-fat milk powder in TBST buffer (15 mM NaCl, 1 mM TRIS-HCl, 0.1% Tween 20, pH 8.0) at room temperature. Each primary antibody (anti-PDGFR-α, anti-PDGFR-β and anti-phosphorylated PDGFR-β, Cell Signaling Technology, Inc. Germany) was diluted in blocking buffer and incubated with the membrane overnight at 4°C. After washing in TBST, the nitrocellulose membrane was incubated with horseradish peroxidase-conjugated antibody in blocking buffer at room temperature for 60 minutes. Antibody binding was determined using the enhanced chemiluminescence detection system according to the manufacturer's instructions. Exposures were recorded on hyperfilms for 10 seconds to 3 minutes.

### Immunocytochemistry

Fibroblasts were cultured as in the western blotting assay. After trypsinization of fibroblasts in flasks, 20 μl cell suspensions (3 × 10^4 ^cells/ml) were seeded on slips and incubated for 24 hour at 37°C with 5% CO_2 _and 95% humidity. After treatments with radiation (10 Gy), SU9518 (5 μM) or SU9518 + radiation (1 hour incubation with SU9518 prior radiation), fibroblasts were incubated 1/2, 6, 12 or 24 hours in MPM. After stimulation fibroblasts were washed with phosphate-buffered saline (PBS) and fixed with methanol and acetone 5 minutes at -20°C respectively. After washing with PBS, fibroblasts were permeabilized for 30 minutes with PBS containing 1% tween-20 (Merck, Darmstadt, Germany), and blocked for 30 minutes with PBS blocking buffer (containing 5% FBS with 1% tween-20). The cells were incubated for 12 hours at 18–20°C in blocking buffer with 3 primary antibodies: anti-PDGFR-α, anti-PDGFR-β and anti-phosphorylated PDGFR-β (Cell Signaling Technology, Inc. Germany). After washing with PBS, the cells were incubated for 1 hour at room temperature with 1:250 secondary antibodies, goat anti-mouse IgG (Alexa Fluor 488) or goat anti-rabbit IgG (Alexa Fluor 488), and mixed 1:200 with bromphenolblue (Sigma, St. Louis, USA) solution for cell nucleus staining. After further washing slides were mounted in Citifluor mounting medium and were examined with a fluorescence microscope (400X, software Openlab 3.1.4, Leica, DMIRB).

### Clonogenic survival assay

To investigate the effects of SU9518 on clonogenic survival of fibroblast and endothelial cells, cells were plated with increasing numbers (10^2 ^to 5 × 10^4^) in 25 cm^2 ^flasks. Cells were incubated with 0–10 μM SU9518 in presence of 10 ng/ml PDGF-AB. Flasks were returned to the incubator for 14–17 days, after which they were stained with crystal violet (Sigma, Germany). Colonies were counted and the surviving percentage was determined for clonogenic survival after correcting for plating efficiency.

### Proliferation assay

Cells were harvested by trypsinization at 37°C and neutralized with trypsin-neutralizing solution. A suspension of 50000 cells in MPM/DMEM was added to 25 cm^2 ^flasks (Becton Dickinson, Heidelberg, Germany). Cells were incubated with SU9518 for 1 hour in cytokine-free medium at standard conditions and incubated for another 72 hours in final mediums (either with 10 ng/ml PDGF-AB, or with 2 ng/ml VEGF + 4 ng/ml bFGF). Cells were then dispersed in trypsin, resuspended, and counted in a coulter counter.

In fibroblasts, PDGF-isoform dependent proliferation assays were performed as follows: Fibroblasts were cultured and plated as in the clonogenic assay. Cells were incubated for 72 hours either with PDGF (-AA, -AB, -BB, each isoform in 2 and 10 ng/ml) alone, or with additional administration of 5 μM SU9518. Cells were then dispersed in trypsin, resuspended, and counted in a coulter counter.

### Co-culture model

A proliferation assay in a co-culture model was used to assess the proliferation ability of cells in the upper compartments after irradiation of cells in the lower compartments with or without SU9518 treatment. Human dermal microvascular endothelial cells (HDMVECs) were grown in 24 well plates. At confluence, the media were changed. Cells were irradiated with 10 Gy using a 6 MeV x-rays linear accelerator at a dose rate of 118 cGy/min. Transwell inserts of upper compartments with a 0.6 μm pore size were plated with 20,000 cells. In SU9518 treated groups and SU9518 + radiation groups, cells in upper compartments were preincubated with 5 μM SU9518 for 1 hour. After selective irradiation of cells in lower compartments, transwells of upper compartments containing cells were transferred into the 24 well plates, incubated for 72 hours at standard conditions, then dispersed in trypsin, resuspended and counted. Three conditions were analyzed: (A) irradiated fibroblast cells in the lower compartment, as the chemotactic "agent" after radiation, and (non irradiated) fibroblasts in the upper compartment; (B) irradiated HDMVEC in the lower compartment and fibroblasts in the upper compartment; (C) irradiated fibroblasts in the lower compartment and non irradiated HDMVEC in the upper compartment.

### Statistical analysis

Statistical analyses were performed with a statistical analysis software system (Statistica 6.0). Student's t test was used to compare means. For multiple comparisons ANOVA was used with Fisher's least-significant difference method. P-values less than 0.05 were considered statistically significant.

## Results

### PDGFR protein regulation in fibroblasts

**Figure 1:**
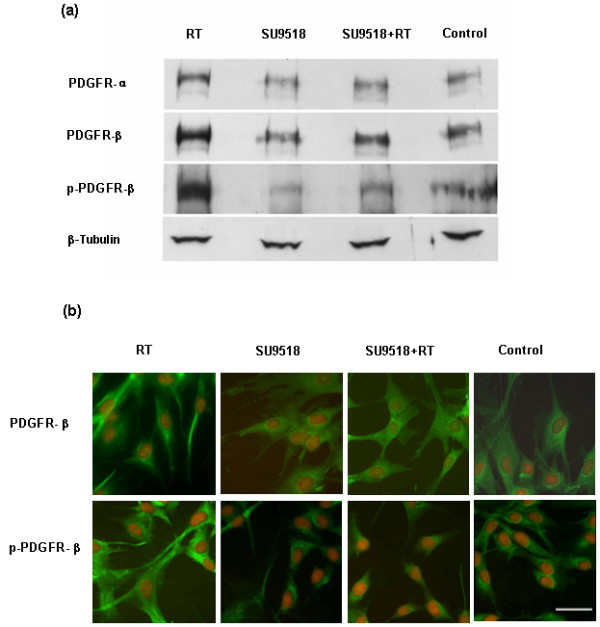
**(a) **Western-blotting analysis of PDGFR-α/-β and phosphorylated PDGFR-β (p-PDGFR-β). Human dermal fibroblast cells were treated with or without 5 μM SU9518 for 1 hour before 10 Gy irradiation. Whole cell lysates of fibroblasts were collected for protein content and separated by SDS-PAGE and transferred to nitrocellulose. The amount of PDGFR and phosphorylated PDGFR was visualized with anti-PDGFR-α, anti-PDGFR-β or anti-phosphorylated PDGFR-β antibodies, respectively. Radiation-induced PDGFR phosphorylation was markedly inhibited by SU9518. **(b) **Immunocytochemistry of human primary dermal fibroblast cells. Fibroblasts were analyzed as untreated (control), after SU9518 (5 μM), irradiation (10Gy) or combined SU9518 and radiation treatment. The cells were incubated 6 hours post radiation and stained with the polyclonal rabbit anti-PDGFR-β/anti-p-PDGFR-β antibody followed by incubation with Alexa Fluor 488 goat anti-rabbit secondary antibody. For nuclear localization the cells were counterstained with propidium jodide (red color). Immunofluorescence staining for PDGFR-β/p-PDGFR-β was indicated by green fluorescence. Irradiation-induced phosphorylation of PDGFR-β and that was substantially inhibited by SU9518. Scale bar presents 25 μm.

To investigate the impact of radiation to PDGFR in fibroblasts and the effect of SU9518 on phosphorylation of PDGF RTK in fibroblasts after radiation, human dermal fibroblast cells were pre-treated with 5 μM SU9518 one hour before 10 Gy irradiation and incubated for 30 minutes (Figure 1a). PDGFR levels as well as PDGFR phosphorylation were determined by western-blotting using primary antibodies specific to PDGFR-α, PDGFR-β or phosphorylated PDGFR-β (p-PDGFR-β), respectively. Irradiation stimulated overexpression of both PDGFR-α and -β in fibroblasts, while expression of PDGFR-β was more upregulated than PDGFR-α. Moreover, irradiation resulted in significantly increased PDGFR-β phosphorylation while SU9518 treatment particularly inhibited phosphorylation of PDGFR-β.

### Immunocytochemistry

**Figure 2:**
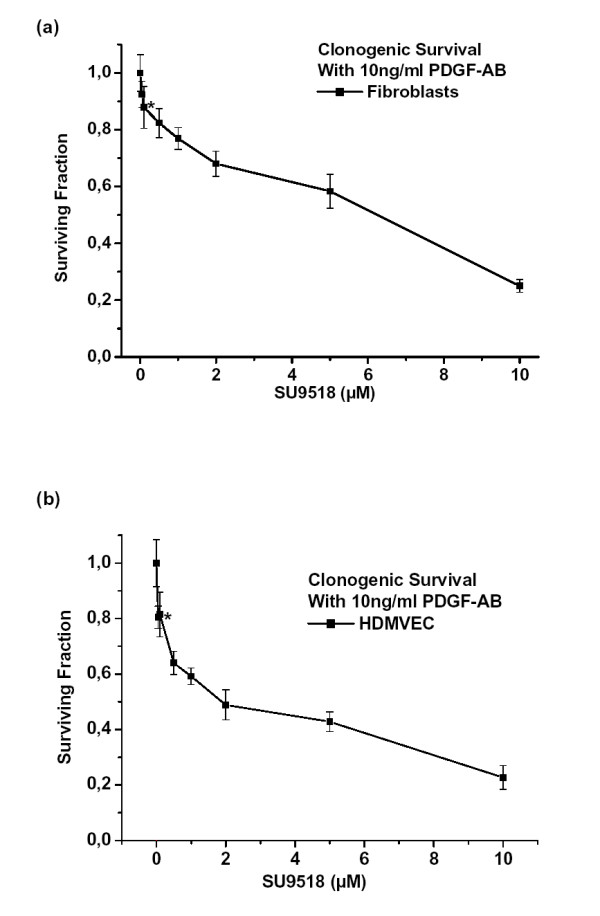
**(a) **Clonogenic survival assay of dermal fibroblasts. Fibroblast cells (10^2 ^to 5 × 10^4^) were incubated with 0–10 μM SU9518 in presence of 10 ng/ml PDGF-AB. Cultures were returned to the incubator for 14–17 days, after which they were stained with crystal violet (Sigma, Germany). Colonies were counted and the surviving percentage was determined for clonogenic survival after correcting for plating efficiency. Bars are means of 5 experiments ± SD. **(b) **Clonogenic survival assay of human dermal microvascular endothelial cells (HDMVECs). Endothelial cells were treated with 0–10 μM SU9518 in the presence of 10 ng/ml PDGF-AB in the media. Surviving fraction was calculated based on the number of colonies counted after 14–17 days. Bars are means of 5 experiments ± SD.

To investigate radiation-induced PDGF signaling in fibroblasts in the presence or absence of SU9518, immunocytochemistry analyses were performed at various time points (1/2, 1, 6 hours) after irradiation. Irradiation resulted in enhanced phosphorylation of PDGFR-β (p-PDGFR-β) in fibroblasts. Representative microscopic photos of fibroblasts with PDGFR-β or p-PDGFR-β staining are presented at time points 1/2 and 6 hours after radiation, respectively (Fig. 2b). Importantly, the autophosphorylation of PDGFR-β was markedly inhibited by 5 μM SU9518, consistent with the results in western-blotting assays.

### Fibroblasts clonogenic survival

**Figure 3:**
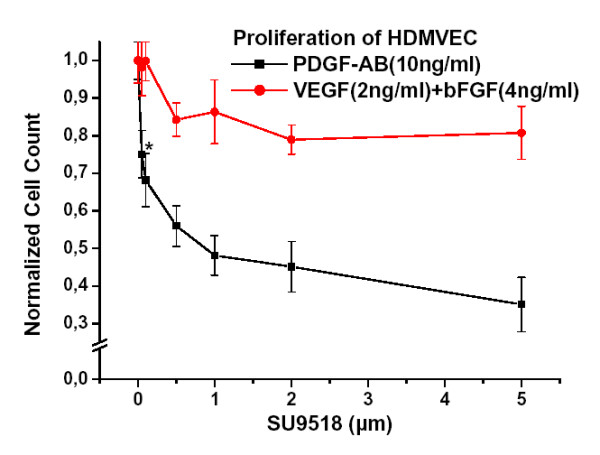
Proliferation assay of HDMVECs. Endothelial cells were plated in collagen I-coated flask and stimulated with 10 ng/ml PDGF (black line) or 2 ng/ml VEGF + 4 ng/ml bFGF (red line) in the absence or presence of SU9518 with increasing concentration (0.1, 0.5, 1, 2 and 5 μM). 72 hours later cells were counted and normalized to respective controls without SU9518.

In the clonogenic survival assay, stimulating effects of PDGF (-AB isoform, 10 ng/ml) on fibroblast cells were examined in the absence or presence of SU9518 with increasing concentrations (Figure 2a). Inhibition of PDGF signaling by SU9518 reduced the clonogenic survival fraction of fibroblasts. The surviving fraction of fibroblasts was reduced by 10 μM SU9518 to 0.23 fold (Figure 3), suggesting that PDGF played a role for the clonogenic survival of fibroblasts which in turn would explain why the small molecular RTK inhibitor SU9518 inhibits fibroblast clonogenic survival.

### Endothelial cell clonogenic survival

In the clonogenic survival assay (Figure 3a), the inhibitory effects of SU9518 on human dermal microvascular endothelial cells (HDMVECs) were examined in the presence of PDGF (-AB isoform, 10 ng/ml). Inhibition of PDGF signaling with SU9518 reduced the survival fraction of endothelial cells, which underscored the importance of the PDGF pathway for the survival of endothelial cells. For example, using 5 μM SU9518, surviving fraction of endothelial cells decreased to 42% and further to 22% at 10 μM SU9518. Thus the PDGF-mediated clonogenic survival of endothelial cells was significantly inhibited by the administration of SU9518 in a dose dependent manner.

### Endothelial cell proliferation

To further evaluate the specificity of SU9518, its ability to inhibit PDGF-stimulated endothelial proliferation was measured and compared with its inhibitory potency to endothelial proliferation stimulated by VEGF and bFGF. We found that SU9518 inhibited 10 ng/ml PDGF-induced endothelial proliferation with an IC50 of approximately 0.9 μM. PDGF-induced proliferation was effectively blocked by SU9518, whereas endothelial proliferation stimulated by VEGF and bFGF was inhibited to a much lesser degree by SU9518 (Figure 2b). For example, the cell number reduction by 1 μM SU9518 was 52% after PDGF stimulation versus 13% after stimulation by VEGF and bFGF.

### PDGF-isoforms dependent fibroblasts proliferation

**Figure 4:**
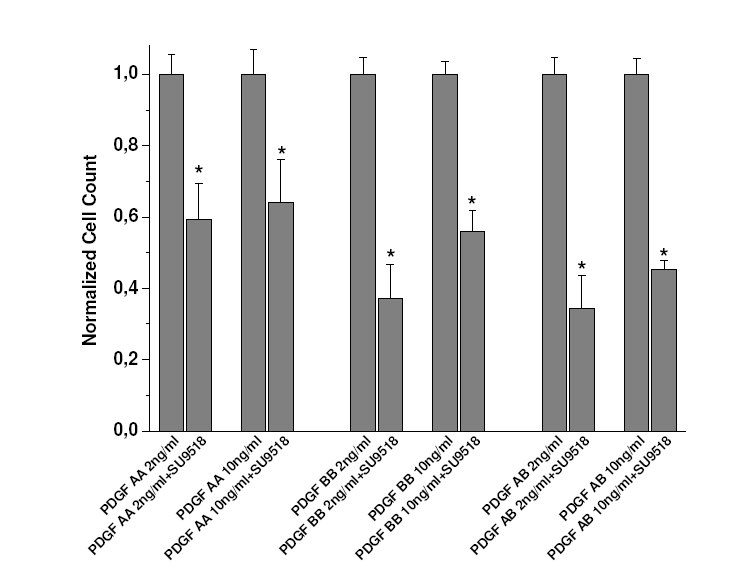
PDGF-isoform dependent fibroblast proliferation assay. Fibroblasts were stimulated by PDGF-isoforms (-AA, -BB and -AB) at 2 ng/ml or 10 ng/ml for 72 hours after 1 hour pre-treatment with or without SU9518. SU9518 inhibited all three isoforms stimulated proliferation of fibroblasts (p < 0.05). The extent of inhibition was greater for PDGF-AB and -BB. Bars are means of 5 experiments ± SD.

Several PDGF-isoforms are found to stimulate different PDGFR subtypes. To further assess the effects of SU9518 we investigated the PDGF-isoforms dependent inhibition of fibroblast proliferation at 2 ng/ml or 10 ng/ml concentrations of various PDGF-isoforms. We found that SU9518 significantly inhibited fibroblast proliferation induced by all three PDGF-isoforms at a concentration of 5 μM (Figure 4). The inhibitory potency of SU9518 to PDGF-AB and -BB appeared to be slightly greater than -AA, while that to PDGF-AB was the most pronounced among the three isoforms.

### Radiation-induced interactions between fibroblasts and endothelial cells

**Figure 5:**
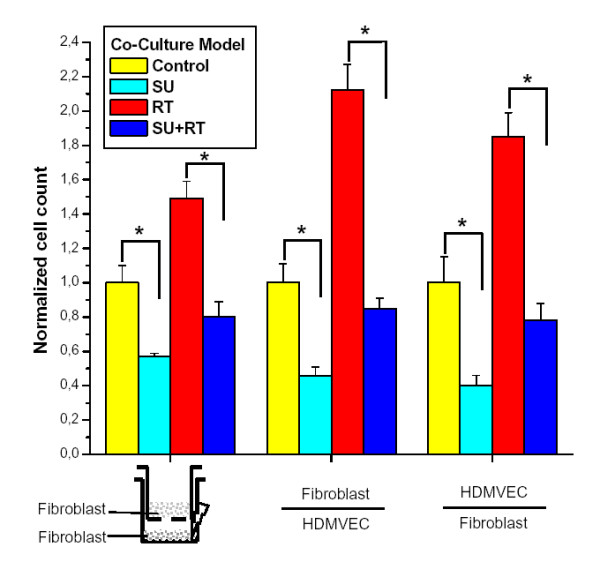
Co-culture experiments. Cells in lower compartments were selectively irradiated to induce paracrine proliferation of cells in upper compartments. Radiation (10Gy) of endothelial cells (HDMVECs) and fibroblast cells stimulated fibroblast proliferation (RT) in upper compartments. The administration of SU9518 significantly inhibited radiation-induced fibroblast proliferation. Likewise, irradiation of fibroblasts stimulated the proliferation of HDMVECs. The paracrine induction of endothelial cell proliferation was significantly inhibited by SU9518. The basal paracrine mitogenic stimulation of fibroblasts or endothelial cells in the upper compartment induced by co-culturing with non irradiated control cells in the lower compartment was also effectively inhibited by SU9518 (all SU9518 vs. control groups). Bars are mean ± SD. In all figures asterisks indicate degree of statistical significance: *significant, P < 0.05; **highly significant P < 0.01.

To investigate the interactions between fibroblasts and endothelial cells a co-culture model was used. Irradiation (10 Gy) of human dermal microvascular endothelial cells (HDMVECs) stimulated paracrine fibroblast proliferation (2.1 fold). Likewise, irradiation of fibroblast cells also stimulated fibroblast cells and endothelial cells in the non irradiated upper compartment up to 1.5 fold (Figure 5). Importantly, the paracrine fibroblast stimulation by either fibroblast or endothelial cells was markedly reduced with the administration of SU9518 by approximately 50% (P < 0.001). Thus SU9518 could significantly inhibit the activation of radiation-induced direct and indirect fibroblast activation.

## Discussion

In the context of fibrosis development in conventional cancer therapy, including radio- and chemotherapy, we studied here the effects of SU9518, a receptor tyrosine kinase inhibitor of PDGF signaling on human primary dermal fibroblasts and endothelial cells. We had previously shown that using this class of molecular targeted compounds – including SU9518 and Imatinib (Gleevec) as well as SU11657 (similar to SU11248, a compound currently in several clinical phase I and II anticancer trials) – can attenuate ionizing radiation-induced lung fibrosis *in vivo *if it is given either prior to or after radiation insult [13]. Here we investigate *in vitro *whether primary human endothelial and fibroblast cells are an important primary target of the RTK inhibitor in the context of PDGF signaling. We found that, *in vitro*, irradiation of fibroblasts and endothelium induced autocrine and paracrine PDGF expression. Functionally, the isolated irradiation of primary human endothelial cells stimulated proliferation in human fibroblasts in a co-culture model. Thus irradiation has effects which can be described as an indirect promotion of fibroblast survival and proliferation. Importantly, RTK inhibitors could effectively attenuate this paracrine radiation-induced activation by blocking autophosphorylation of PDGFR and by downregulation of PDGFR expression. Since fibroblasts are most likely key players in radiotherapy related fibrosis, and given the close correlation between PDGF signaling and fibroblasts activity (e.g. survival and proliferation), along with results in vivo [13] we conclude that fibroblasts may as well be a target of RTK inhibitors in the process of attenuating radiation-induced fibrogenesis in vivo.

This conclusion is in agreement with earlier reports showing that PDGF is a potent mitogen and chemoattractant for mesenchymal cells and also a chemoattractant for neutrophils and monocytes [16-18]. Similarly, several studies had reported that PDGF isoforms and their receptors were upregulated during pathologic fibroproliferative diseases in humans and in murine models by different types of impairment (e.g. radiation- [19,20]/bleomycin- [11] induced fibrosis, idiopathic pulmonary fibrosis [21]) vindicating the importance of the PDGF/PDGFR system in proliferative diseases.

PDGFR-β has been suggested to be predominantly expressed in fibroblasts [12] in agreement with our data presented here. In addition, we found that PDGFR-β was more upregulated after radiation in fibroblasts compared with PDGFR-α in both western blotting and immunocytochemistry assays. Others had shown that fibroblasts isolated from rat lungs over expressed PDGFR-β with higher chemotactic response to PDGF-BB in vivo [20]. Although the ultimate mechanism of PDGFR overexpression remains unclear, we found ionizing radiation can upregulate PDGFR in fibroblasts. More importantly, for the ultimate goal of treating or preventing fibrosis in patients, we found that SU9518 attenuated the radiation-induced upregulation of PDGFR.

The best characterized mechanisms by which PDGF down-streaming signaling mediates cellular responses involve the activation of the ras/MAPK pathway, which can functionally increase cellular proliferation, and the PI3k/Akt pathway, which promotes cell survival in general tumor biology [22]. PDGF has also been shown to stimulate mitogenicity and chemotaxis of fibroblasts and smooth muscle cells [12]. In our fibroblast clonogenic and proliferation assays, inhibition of PDGF signaling by SU9518 significantly reduced the clonogenic survival fraction and the proliferation of fibroblasts, thus supporting the important role of PDGF signaling for fibroblasts survival. Moreover, PDGF has been shown to stimulate the production of several matrix molecules, such as collagen, fibronectin, and proteoglycan [23-25]. The resulting close correlation between PDGF/PDGFR and fibroblasts may explain the inhibitory effect of SU9518 to radiation-induced fibrosis [13].

To further evaluate the specificity of SU9518, its ability to inhibit cell proliferation stimulated by PDGF (-AB isoform) or combined VEGF and bFGF was measured, because these growth factors have been shown to induce similar signaling pathways, changes in gene expression, and cellular responses in mesenchymal cells [26].

Unresolved issues in fibrosis research inlclude the relative contribution of different cell types to the fibrotic development and the usefulness of acute anti-inflammatory drugs such as corticosteroids. A Cochrane review of corticosteroids in idiopathic lung fibrosis found no evidence that steroid treatment was of any benefit, in spite of repression of early inflammation [27]. Savikko et al demonstrated that cyclosporin A treatment cannot inhibit the expression of PDGF ligands and receptors, although it ameliorated the extent of inflammation [28]. This suggests the involvement of other cells besides acute inflammatory cells in the development of fibrosis. In vivo in lungs, the radiation-induced fibroproliferative process occurs within the microenvironment of the distal gas exchange units consisting of fibroblasts, epithelial cells [29,30] and endothelial cells [31]. In agreement with our co-culture results, endothelial cells are described as potential sources of PDGF after radiation [6]. Epithelial cells do not express PDGF and PDGFR under physiological conditions. But expression of c-sis mRNA in epithelial cells has been reported in certain pathologic fibrotic states, such as in patients with idiopathic lung fibrosis [21].

SU9518 exerts its inhibitory effect by ATP-competitive inhibition of the catalytic activity of the tyrosine kinase after PDGFR activation [32]. Under normal conditions PDGF-AA induces α/α receptor dimers, PDGF-AB induces α/α and α/β receptor dimers and PDGF-BB induces all three receptor dimer combinations. Since SU9518 inhibited fibroblast proliferation activated by all three isoforms, it is conceivable that SU9518 can inhibit both PDGFR-α and -β.

Despite many investigations, the relative roles of most cytokines/growth factors in fibroproliferative diseases are not completely defined. For some of the putative key players such as TGF-β, IL-1, TNF-α and thrombin it has been reported that they may exert their profibrotic activities through a PDGF-dependent pattern [33-36]. One should keep in mind however that in the case of ionizing radiation the signaling cascades in cells are complex and the resulting regulations may well be described as a network of signaling [36-40]. Nevertheless, PDGF may be an important node in this network and thus a key cytokine responsible for the fibrotic development, suggesting that perturbation of the PDGF/PDGFR system could provide an effective means of inhibiting fibrosis.

## Conclusion

Radiation-induced autocrine and paracrine PDGF signaling plays an important role in fibroblast and endothelial cell activation and proliferation. PDGFR tyrosine kinase inhibition reduces radiation-induced fibroblast activation. This data supports the notion that SU9518 might exert its antifibrotic activity in vivo via attenuation of PDGF signaling. These results warrant further studies investigating how tyrosine kinase inhibitors with anti-PDGF property may attenuate cancer therapy related fibrosis in addition to their primary antitumoral activity.

## Competing interests

The author(s) declare that they have no competing interests.

## Authors' contributions

ML participated in performing experiments, in data analysis and in writing the manuscript. PG performed western blots and immunocytochemistry. CP participated in writing the manuscript. KEL participated in designing the study and in writing the manuscript. RK, KH and JD participated in writing the manuscript. TT performed the cell assays. AA and PEH designed the study, participated in data analyses and in writing the manuscript. All authors edited and acknowledged the final version of the manuscript.

## Pre-publication history

The pre-publication history for this paper can be accessed here:
